# Using the Health Belief Model to Assess the Physical Exercise Behaviors of International Students in South Korea during the Pandemic

**DOI:** 10.3390/healthcare11040469

**Published:** 2023-02-06

**Authors:** Peng-Shuai Ma, Wi-Young So, Hyongjun Choi

**Affiliations:** 1 Department of Physical Education, Dankook University, Yongin 16890, Republic of Korea; 2 Sports Medicine Major, College of Humanities and Arts, Korea National University of Transportation, Chungju-si 27469, Republic of Korea

**Keywords:** COVID-19, health belief model, international students, physical exercise

## Abstract

International students have the special status of being isolated in a foreign country during a pandemic. As Korea is a worldwide leader in education, it is important to understand the physical exercise behaviors of international students during this pandemic to assess the need for additional policies and support. The health belief model was used to score the physical exercise motivation and behaviors of international students in South Korea during the COVID-19 pandemic. In total, 315 valid questionnaires were obtained and analyzed for this study. The reliability and validity of the data were also assessed. For all variables, the values for combined reliability and the Cronbach’s α were higher than 0.70. The following conclusions were drawn by comparing the differences between the measures. The results of the Kaiser–Meyer–Olkin and Bartlett tests were also higher than 0.70, confirming high reliability and validity. This study found a correlation between the health beliefs of international students and age, education, and accommodation. Consequently, international students with lower health belief scores should be encouraged to pay more attention to their personal health, participate in more physical exercise, strengthen their motivation to participate in physical exercise, and increase the frequency of their participation.

## 1. Introduction

As a top-ranking country among the Global Citizens for Human Rights’ World’s Best Education Systems [1], South Korea has been recognized worldwide for the comprehensiveness and quality of its education system. Hence, over the last decade, the number of international students in Korea has increased. In December 2021, this number reached 152,281, an increase of 44.9% over the total number in 2010 [2]. However, the coronavirus has had an impact on these numbers, as well as on the lives of these international students in Korea.

The coronavirus disease 2019 (COVID-19) was declared a global pandemic on 12 March 2020, by the World Health Organization [3]. Therefore, in the context of COVID-19, over the last few years the number of international students coming to South Korea has decreased. In 2020, the number decreased by 6470 compared with that in 2019, and the number in 2021 decreased by 1414 compared with that in 2020 [2]. With the end of the epidemic, the number of students studying abroad has increased by 14,611 compared to 2021 [2]. During this time, most countries imposed restrictions to reduce the spread of COVID-19; for example, these included restrictions on indoor and outdoor gatherings, business hours, quarantine periods, and international travel [4]. These restrictions have had many effects, including a reduction in physical activity. Due to the emergence of COVID-19, international students in South Korea have fewer opportunities to go out to participate in sports activities. In such circumstances, students’ physical health status may change. This is because social interaction behaviors that occur when going out to participate in physical exercise positively impact the mental health of international students. Thus, reduced physical exercise may lead to negative changes in the mental health of international students, which will also negatively impact their quality of life [5].

Physical exercise has been shown to be an important element in maintaining one’s physical, as well as mental, health [5,6]. Physical activity and exercise might be key factors in helping the population better tolerate pandemic periods at both the mental and physical levels [7]. During the pandemic, participation in such activities changed, as physical exercise activities in most countries and regions around the world were affected [7,8]. This was particularly true in South Korea. During the pandemic, Korean citizens’ participation in sports gradually shifted from outdoors to indoors, and from clustered multi-person sports to single-person non-clustered sports [8]. For an international student unfamiliar with the Korean lifestyle and the surrounding environment, physical exercise during the COVID-19 pandemic became difficult.

Regular physical exercise not only improves the body’s immunity [5], but also effectively reduces disease fatality rates [6]. The health belief model (HBM), first proposed by American psychologist Rosenstock over six decades ago, assesses health-related attitudes and behaviors. It has been widely used, modified, and improved [9]. The model is a practical tool that can be applied to participation in physical exercise to improve our understanding of the underlying influential factors [10].

The HBM evaluates the health status of individuals in terms of their health beliefs, cues, or intentions, and behavioral constraints [11]. This research mainly focuses on five factors: perceived benefits of exercise, self-efficacy of physical evaluation, susceptibility to disease, severity of sensory illness and frailty, and paying attention to the results of physical evaluation. Although physical exercise is a good means of improving health, many people do not participate in it; thus, a better understanding of this phenomenon will enable us to promote better participation behaviors [11,12]. We use the HBM in our study to assess the health beliefs of international students and investigate how these beliefs correlate with physical activity during the pandemic.

Previous studies have found that health beliefs can help people take effective preventive measures. Therefore, establishing good health beliefs has a certain impact on physical exercise motivation and behavior. Physical exercise motivation is generally divided into physical health, appearance, entertainment, social communication, and other aspects [13]. However, groups with different educational levels and social backgrounds also have different health beliefs. People with higher educational backgrounds may have more advantages in the process of establishing personal health beliefs [14], as they have richer knowledge reserves and more life experience. Their personal health beliefs may be more comprehensive [13,15]. The environment may be a factor as well. In South Korea, international students can choose whether to live on campus or off campus, which also has an important impact on their lifestyles. Students living off campus may be more physically active than those living on campus [16,17].

At present, there are many studies on the physical exercise of college students in the context of COVID-19, but few focusing on the international student group [5,9,17,18,19,20]. We investigate the correlations between the health beliefs of international students, their education level, physical activity, and their living situation before and after COVID-19. Based on the importance of physical activity for a student’s mental health, this study aims to provide a reference for students, academia, and policymakers regarding the motivation and willingness of international students to participate in physical exercise during the normalization of the pandemic. To that end, we use the HBM to understand students’ awareness of their health and assess the impact of their scores on their participation in physical exercise in South Korea.

## 2. Materials and Methods

### 2.1. Data Collection

As international students at Dankook University, Yongin, South Korea, we created an online questionnaire through the website www.wjx.cn, accessed on 12 August 2021. Through our teachers and friends, we sent the questionnaire’s link to the surrounding foreign students. To explore physical exercise behavior and changes in such behavior during the COVID-19 pandemic in the context of the HBM, on 3 March 2022, an online questionnaire was used to maintain social distancing, and students in the Seoul and Gyeonggi-do regions of South Korea were randomly selected to participate. A survey of 320 foreign students studying in South Korea used the HBM to assess their health behaviors and changes in their physical exercise behaviors. All 320 online questionnaires distributed were returned. Of these, 315 were valid, which led to an effective response rate of 98.40%. Since data sets on this study from the online questionnaire did not include private identifier information, such as telephone numbers, home addresses, and social security numbers, ethical approval was not required. Ultimately, the study was approved by the institutional review board of Dankook University, Yongin, South Korea, and ethical approval was waived.

### 2.2. Scale Design

There are relatively complete measurement scales in the extant research for the HBM [18]. This study transformed the HBM scale created by DAI Xia, deleted the inappropriate variables, and adapted them according to the specificity of the COVID-19 context. We based the measurement scale in our study on similar variables in previous research with adequate content credibility [12,16,18,21]. The questions were modified and adjusted accordingly, as shown in Table 1. All the items on the scale were measured against a five-point Likert scale, where 1 meant “strongly disagree” and 5 meant “strongly agree.” At the same time, a score from 1 to 5 was assigned to the questionnaire responses on cognitive health belief patterns in this survey (only perceived benefits of exercise, self-efficacy of physical evaluation, susceptibility to disease, severity of sensory illness and frailty, and paying attention to the results of physical evaluation).

### 2.3. Data Analysis

To study the HBM from the perspective of international students in South Korea and their physical exercise behaviors, we included the HBM’s questions as well as questions regarding the differences in student motivation to participate in physical exercise and students’ actual exercise behaviors before and after the pandemic. We also conducted several tests to ensure the reliability and validity of our variables. The data of the Internet survey were organized. SPSS 26.0′s (IBM Corp., Armonk, NY, USA) Kaiser–Meyer–Olkin (KMO) Test was used to verify the validity of the results. In order to understand the importance attached to health, appearance, entertainment, learning skills, and social interactions under different educational backgrounds, the data were compared and sorted by analysis of variance and least square difference (ANOVA-LSD) post-hoc tests. The statistical significance was set at *p* < 0.05.

## 3. Results

### 3.1. Reliability and Validity

The combined reliability and Cronbach’s α of most variables were higher than the recommended level of 0.70, and the KMO and Bartlett test results were higher than 0.70, thus assuring high validity. The reliability and validity test results are shown in Table 1.

To accurately understand the differences in each group’s physical exercise behaviors and motivation in terms of education and other factors, we used the ANOVA-LSD to analyze the differences assuming a variance.

### 3.2. Sociodemographic Variable Analysis

Among the participants in this survey, international students from China accounted for the majority (69.84%), followed by those from Vietnam (22.54%). The number of international students from other countries and regions was relatively small (7.61%). Most of the international students were female (61.59%), between 23–25 years old (41.27%), and in either their third or fourth semester (52.06%). International students over 23 years old in at least their second semester can adapt to the surrounding environment of the school. Most international students (60.32%) chose more free off-campus accommodations. Details of the sociodemographic variables are shown in Table 2.

### 3.3. Correlations among Age, Gender, and Health Beliefs

To understand the differences among international students in the context of the HBM across different age groups, we first tested the variables using Pearson correlation analysis in SPSS. The test results are shown in Table 3.

Among the international students, the benefits of activity significantly correlated with age (*p* < 0.01), education (*p* < 0.01), and lifestyle (*p* < 0.01). Both male and female students with increased health awareness perceived the benefits of exercise and enjoyed it more [22]. Our tests regarding differences in awareness based on health beliefs among international students in different age groups indicated that higher HBM scores correlated significantly with age, gender, educational background, and lifestyle among the international students surveyed. Age was closely related to personal life experience and perception of ontological health, and those with extensive experiences were more conscious of their health [23].

**Figure 1:**
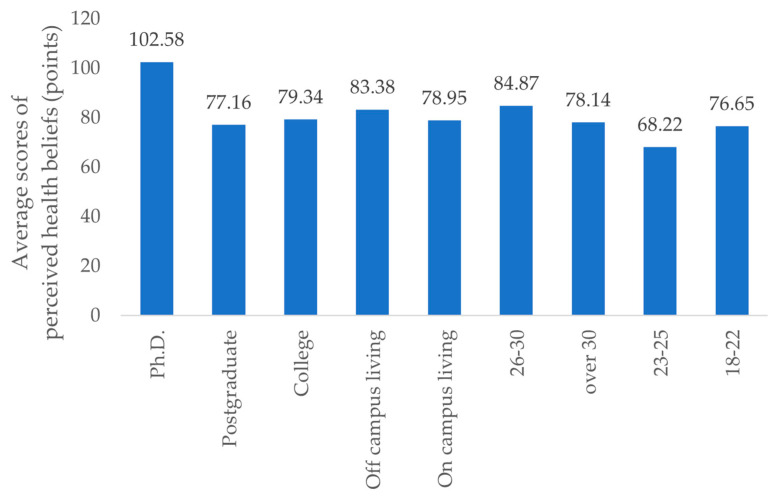
The average scores of perceived health beliefs for each group.

Figure 1 presents the average scores of perceived health beliefs for groups. After re-organizing the HBM’s score data from the questionnaire survey for different educational backgrounds, accommodations, and age groups, we found no significant correlations regarding comprehensive HBM scores for the vast majority of international students by gender. Those with higher average HBM scores were more educated, 26–30 years old, and lived off campus. Older international students with more education appeared to have increased safety awareness when living alone [18].

### 3.4. Correlations of Physical Exercise Motivation and Education

In Table 4, through the ANOVA-LSD analysis of academic qualifications, we found that following the pandemic outbreak, foreign undergraduate students had a stronger desire to improve their health, maintain their bodies, and enjoy sports, as well as a stronger need for social interaction, than did graduate and doctoral students. Thus, the sudden pandemic situation had a greater impact on the health motivation of international students with less education. Through the same analysis in terms of age difference, the motivation for physical exercise among 26–30-year-olds was lower than that among the other age groups. Although older international students had stronger personal health awareness, they perceived that the pandemic made it more difficult for them to get physical exercise. Motivation was less affected among the other age groups. In terms of living situation, students living off campus reflected a smaller difference in physical exercise motivation than did those living on campus. With the spread of the pandemic, more people chose to exercise at home. Students living off campus have more favorable conditions for such exercise.

### 3.5. Influence of HBM Scores on International Students’ Physical Exercise Motivation and Behavior

**Figure 2:**
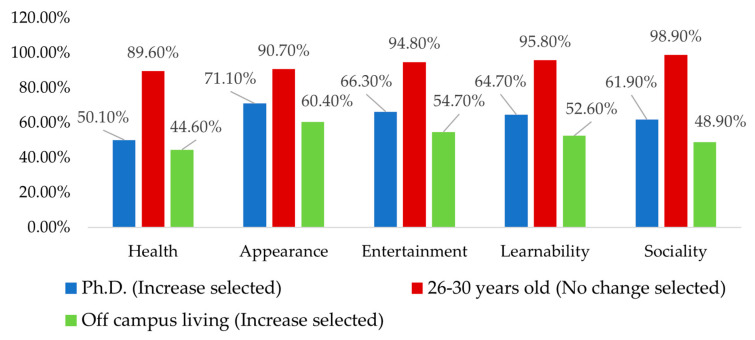
The exercise motivation intention factors with the highest average health belief scores.

Figure 2 shows how the average HBM scores differ based on educational background, age, and living situation. Those with higher average scores were aged 26–30 years (84.87 points), had doctoral degrees (102.58 points), and had off-campus accommodations (83.38 points). Changes in motivation for training and exercise were also significantly different in the group of international students with higher health belief scores. Among them, students with doctoral degrees were more inclined to choose the “increase” option after the outbreak of the pandemic. The international students in the 26–30-year-old group chose the “unchanged” option, and the students living off campus chose the “increase” option.

In Table 5, the options from “Physical exercise behavior differences” questionnaire were assigned to the participants’ corresponding scores of the questionnaire concerning the most exercise behaviors (except for the ninth question). Next, the average score was calculated for each group. In terms of physical exercise activities, the overall exercise intensity of the international students with doctoral degrees between the ages of 26–30 years was moderate, and their exercise time was generally 31–59 min about 5–6 times a week. Their exercise intensity did not change after the pandemic. However, in terms of physical exercise behavior, the average score for international students aged 26–30 years was 79.2 points, which was higher than that for those with doctoral degrees, at 59.69 points. The average score for international students living off campus was the lowest, at only 58.1 points. However, we also found that after the outbreak of the pandemic, international students living off campus changed their answers from “sweating a lot, vigorous but not lasting exercise (such as playing basketball, tennis, etc.)” to “shortness of breath, sweating a lot, intense and long-lasting exercise (such as long-distance running, playing badminton, swimming, etc.)”, implying that their exercise intensity increased somewhat.

## 4. Discussion

At present, there are many studies on college students’ exercise within the context of the COVID-19 pandemic, but few studies focus on the international student group. International students faced a particularly difficult situation when the pandemic occurred because of their special status as foreigners. There are relatively few channels for understanding and interpreting the local pandemic prevention and control policies. Therefore, it is necessary to study international students’ physical exercise behaviors during the pandemic [18,24].

This study investigated the HBM in relation to physical exercise motivation and behavior among 315 international students in South Korea within the context of the pandemic. The average health belief scores among international students with doctoral degrees, those aged 26–30 years, and those who lived in off-campus housing were higher than those of students with other educational backgrounds, in other age groups, and those who lived on campus. The motivation and behavior of international students with high average health belief scores did not change much before and after the pandemic. This shows that overseas students with higher academic qualifications, of a moderately mature age, and who live off campus in a more free-living environment have strong health beliefs. Their abilities to perceive and manage their bodies’ health status are strong, and they have relatively regular physical exercise habits.

Previous studies have found that COVID-19 health beliefs can help people take effective preventive measures [25,26]; therefore, the establishment of good health beliefs has a certain impact on physical activity motivation and behavior. Physical exercise motivation is generally divided into physical health, appearance, entertainment, social communication, and other aspects [14,21,27]. Perceived exercise benefits allow participants to feel the physical benefits of exercise while performing it, so that people enjoy exercise. Physical fitness evaluation self-efficacy helps people to effectively recognize the relationship between physical health and exercise, so that exercise participants can reasonably arrange physical exercise according to their own health status. When someone is in poor physical health, there will also be concerns about and associations with physical illness. On the other hand, people’s positive attitudes may also stimulate them to actively participate in sports. Since most people have no experience of being infected or sick with COVID-19, their ability to perceive the threat of the disease is poor. Therefore, their motivation to participate in preventive physical exercise will also be weakened; paying attention to the results of physical health evaluation is the fastest way to understand the physical health status of individuals, and it is also a more direct factor affecting physical exercise motivation and behavior.

However, groups with different educational levels and social backgrounds also have different health beliefs. People with higher educational backgrounds have more advantages in the process of establishing personal health beliefs [27,28], as well as richer knowledge reserves and life experience. This is consistent with the results of this study. This study also analyzed the correlations between the interviewees’ educational background and age and found that for international students with more education and of a relatively advanced age, the establishment of the personal HBM is also more complete. People around the age of 30 have a strong awareness of health and physical exercise, and they have a relatively regular lifestyle. This is very helpful for maintaining physical and mental health [14,29,30]. In South Korea, students can choose whether to live in on-campus dormitories, and both on-campus and off-campus dormitories also have an important impact on students’ lifestyles. In China, college students are not allowed to live off campus, so their lifestyles are relatively simple. South Korea’s relatively free lifestyle brings more convenience to students’ lives. For example, students can go to gyms to participate in physical exercise, and students living off campus are more active in physical activities than students living on campus [13,20]. In terms of intensity, frequency, and time of physical exercise, due to the impact of the pandemic, most people showed an overall downward trend. This has a stronger relationship with the inability to go out to participate in or enter a gym for physical exercise.

Within the context of the COVID-19 pandemic, the health beliefs of international students in South Korea have a certain influence on their physical exercise motivation and behavior. This, in turn, has a significant impact on physical exercise intensity, exercise frequency, exercise time, and activity range. This study did not investigate respondents’ access to personal health information, so there are some limitations. In the future, we can learn about the specific sports and fitness status of international students by investigating how they obtain information about their health beliefs. In order to study the impact of the HBM on the health status of international students, international students should be assisted in establishing personal health beliefs and maintaining a good level of physical health.

## 5. Conclusions

This study surveyed 315 international students in Seoul and Gyeonggi-do, South Korea. Through the HBM, differences in physical exercise motivation and behavior before and after the pandemic were studied. Our conclusions are as follows: First, during the pandemic, physical exercise motivation among international students with higher health belief scores did not change significantly. This was closely related to the positive physical exercise habits formed through personal behaviors.

Second, the group health belief scores of foreign students with doctoral degrees, who were aged 26–30 years, and who lived off campus were higher than those of other groups. The higher the health belief score, the higher the awareness and concern regarding the student’s personal health status. Third, groups with higher health belief scores showed only small differences in terms of exercise intensity, frequency, time, and scope before and after the pandemic, reinforcing the benefits of strong health beliefs for establishing a strong health awareness and motivating regular physical activity. International students with lower health belief scores should be encouraged to pay more attention to their personal health, participate in more physical exercise, strengthen their motivation to participate in physical exercise, and increase the frequency of their participation.

## Figures and Tables

**Table 1:** The reliability and validity test results for each factor (*n* = 315).

Variables	AVE	CR	Cronbach’s α	KMO & Bartlett
Perceived benefits of exercise	0.942	0.697	0.919	0.936
Self-efficacy of physical evaluation	0.518	0.808	0.721	0.945
Susceptibility to disease	0.868	0.687	0.811	0.919
Severity of sensory illness and frailty	0.745	0.921	0.892	0.938
Paying attention to the results of physical evaluation	0.828	0.935	0.910	0.921
Differences in motivation to participate in physical exercise before and after the emergence of COVID-19	0.611	0.916	0.885	0.926
Physical exercise behavior differences	0.680	0.922	0.802	0.833

Note: AVE, average variance extracted; CR, composite reliability; KMO, Kaiser–Meyer–Olkin.

**Table 2:** Descriptive statistics for the respondents (*n* = 315).

Variables	Category	Frequency	Percentage (%)
Gender	Male	121	38.41
	Female	194	61.59
Age	18–22	52	16.51
	23–25	130	41.27
	26–30	97	30.79
	Over 30	35	11.11
Educational level	College	102	32.38
	Postgraduate	112	35.56
	PhD	101	32.06
Semester	1–2	69	21.90
	3–4	164	52.06
	5–6	60	19.05
	7–8	22	6.98
Living accommodation	On campus	125	39.68
	Off campus	190	60.32

**Table 3:** Pearson correlation analysis of age, gender, and health beliefs (*n* = 315).

Variables	Contents	Sex	Age	Education	Living Situation
Perceived benefits of exercise	I think physical exercise can enhance physical health.	0.086	0.678 **	0.561 **	0.252 **
	I think physical activity can prevent disease and prolong life.	0.109	0.557 **	0.452 **	0.254 **
	I think physical exercise is good for the mind and body.	0.109	0.594 **	0.483 **	0.230 **
	I think physical exercise can keep me fit.	0.097	0.503 **	0.412 **	0.210 **
	I think physical activity improves quality of life.	0.064	0.776 **	0.640 **	0.277 **
	I think physical exercise is an important way to maintain physical health.	0.079	0.496 **	0.419 **	0.235 **
	I think enhancing physical health requires regular physical exercise.	0.130 *	0.551 **	0.469 **	0.267 **
Self-efficacy of physical evaluation	When my physique is in poor health, I feel a sense of urgency to exercise.	0.053	−0.041	0.039	0.098
	When my physique is not in good health, I will work out against the odds.	0.092	0.614 **	0.454 **	0.239 **
	When my physique is not healthy, I can overcome obstacles such as weather and the environment to train.	0.150 **	0.574 **	0.441 **	0.246 **
	Even when I get tired, I will follow a plan to exercise to enhance my physique and health.	0.051	0.487 **	0.383 **	0.177 **
Susceptibility to disease	People with poor physical health are more likely to be infected with COVID-19.	0.126 *	0.284 **	0.202 **	0.205 **
	Lack of exercise may lead to COVID-19 infection.	0.102	0.287 **	0.326 **	0.203 **
	Poor physical health made me worry about contracting COVID-19.	0.234 **	0.353 **	0.320 **	0.249 **
Perceived severity of illness and frailty	Serious investigations of perceived sickness and frailty—I am afraid of contracting COVID-19.	<0.001	−0.048	−0.020	−0.049
	Every time I get sick, I feel scared.	0.069	0.792 **	0.656 **	0.348 **
	I feel a serious threat to my physical health when I have an illness.	0.060	0.654 **	0.584 **	0.297 **
	Lack of exercise can lead to a serious illness.	0.141 *	0.478 **	0.516 **	0.292 **
Paying attention to the results of physical evaluation	Physical health evaluation is an important way for me to understand health.	0.091	0.489 **	0.506 **	0.283 **
	I am very concerned about changes in physical health information.	0.086	0.444 **	0.446 **	0.233 **
	The results of a physical health evaluation allow me to know more about my own health.	0.069	0.792 **	0.656 **	0.348 **

Note: * *p* < 0.05, ** *p* < 0.01.

**Table 4:** Differences in foreign students’ physical exercise motivation based on academic qualifications.

Variables	Education	N	Mean	Standard Deviation	*F*	Least Significant Deviation
Health	Undergraduate	102	4.33	0.569	65.815 ***	1 > 2 > 3
	Masters	103	4.06	0.683		
	Ph.D.	110	3.43	0.515		
Appearance	Undergraduate	102	3.96	0.195	62.739 ***	1 > 2 > 3
	Masters	103	3.81	0.397		
	Ph.D.	110	3.39	0.490		
Entertainment	Undergraduate	102	3.86	0.446	40.888 ***	1 > 2 > 3
	Masters	103	3.76	0.474		
	Ph.D.	110	3.29	0.548		
Learning skills	Undergraduate	102	3.80	0.546	30.639 ***	1 > 2 > 3
	Masters	103	3.74	0.504		
	Ph.D.	110	3.28	0.544		
Social interaction	Undergraduate	102	3.75	0.624	27.439 ***	1 > 2 > 3
	Masters	103	3.71	0.517		
	Ph.D.	110	3.23	0.569		

Note: *** *p* < 0.001; tested using analysis of variance. 1: Undergraduate, 2: Masters, 3: Ph.D.

**Table 5:** Summaries of physical exercise behaviors among those with strong health beliefs before and after the pandemic.

Variables	Exercise Intensity (B)	Exercise Intensity (A)	Exercise Time (B)	Exercise Time (A)	Exercise Frequency (B)	Exercise Frequency (A)	Range (B)	Range (A)
PhD.	High strength (52.2%)	High strength (52.0%)	31–59 min (70.0%)	31–59 min (64.4%)	5–6 times (65.5%)	5–6 times (56.6%)	501–1000 m (65.5%)	501–1000 m (53.3%)
26–30 years old	High strength (77.3%)	High strength (78.3%)	31–59 min (85.5%)	31–59 min (85%)	5–6 times (84.5%)	5–6 times (79.3%)	501–1000 m (81.4%)	501–1000 m (61.8%)
Off-campus living	Medium strength (33.5%)	High strength (34.0%)	31–59 min (65.4%)	31–59 min (62.7%)	5–6 times (73.9%)	5–6 times (69.6%)	501–1000 m (71.8%)	501–1000 m (50.5%)

## Data Availability

The data presented in this study are available upon request from the authors. Some variables were restricted to preserve the anonymity of study participants.
